# *Drosophila melanogaster* in nutrition research—the importance of standardizing experimental diets

**DOI:** 10.1186/s12263-019-0627-9

**Published:** 2019-02-01

**Authors:** Kai Lüersen, Thomas Röder, Gerald Rimbach

**Affiliations:** 1 Institute of Human Nutrition and Food Science, University of Kiel, 24118 Kiel, Germany; 2 Department of Molecular Physiology, Institute of Zoology, Kiel University, Kiel, Germany; 3 Airway Research Center North, German Center for Lung Research (DZL), Kiel, Germany

**Keywords:** *Drosophila melanogaster*, Model organism, Diet, Phenotype, Disease

## Abstract

The fruit fly *Drosophila melanogaster* has been increasingly recognized as an important model organism in nutrition research. In order to conduct nutritional studies in fruit flies, special attention should be given to the composition of the experimental diets. Besides complex diets, which are often based on maize, yeast, sucrose, and agar, *Drosophila* can be also fed chemically defined diets. These so-called holidic diets are standardized in terms of their macro- and micronutrient composition although the quantitative nutrient requirements of flies have yet not been fully established and warrant further investigations. For instance, only few studies address the fatty acid, vitamin, mineral, and trace element requirements of fruit flies. *D. melanogaster* may be also of interest in the field of nutritional medicine. Diet-induced diabetes and obesity models have been established, and in this context, often, the so-called high-fat and high-sugar diets are fed. However, the composition of these diets is not sufficiently defined and varies between studies. A consensus within the scientific community needs to be reached to standardize the exact composition of experimental complex and holidic diets for *D. melanogaster* in nutrition research. Since *D. melanogaster* is an established valuable model system for numerous human diseases, standardized diets are also a prerequisite to conduct diet-disease interaction studies. We suggest that a comprehensive approach, which combines deep phenotyping with disease-related *Drosophila* models under defined dietary conditions, might lead to the foundation of a so-called fly clinic.

## Background

The quality of nutritional studies largely depends on the research question addressed, the experimental design, the statistical power, and the composition of the experimental diets. The vast majority of nutritional studies in model organism have been conducted in laboratory rodents such as mice and rats. Nutrient requirements for rodents are relatively well established including energy, lipids, fatty acids, carbohydrates, proteins, and amino acids as well as vitamins, minerals, and trace elements [1].

The fruit fly *Drosophila melanogaster* has been extensively used as a robust model organism in genetics, developmental biology, aging, and other areas of biomedical research over a long period of time. Only recently experimental nutritionists have begun to consider *Drosophila* as a versatile model organism in food and nutrition research [2]. Thus, it is not surprising that dietary requirements for flies have yet not been fine-tuned to the same extent as for laboratory rodents. As far as complex *Drosophila* diets are concerned, it is interesting to note that many different recipes for complex media have been described in the literature.

In this review, we critically survey the variety of diets—including the preliminary state of chemically defined diets—employed in *Drosophila* research. Moreover, we point out that a standardized diet will be necessary to implement the fruit fly as a promising model organism in diet-disease interaction studies.

### Experimental diets in *Drosophila* research

*Drosophila* diets are often formulated on the basis of yeast, maize, sucrose, and agar [3, 4]. However, the nutrient composition can vary substantially among these recipes. Moreover, sometimes, other ingredients including glucose, barley, soya, peptone, and banana are used. Diets may also differ in terms of preservatives to prolong stability and shelf-life. Most recipes include both p-hydroxy-benzoic acid methyl ester (nipagin) and propionic acid; however, others use solely one of these preservatives, while in some cases, antibiotics such as penicillin-streptomycin or a phosphoric-propionic acid mix are added [3, 4]. Furthermore, also the so-called high-fat and/or high-sugar diets are applied in *D. melanogaster* to induce diabetic or obese phenotypes. However, the composition of “high-fat” or “high-sugar” diets is not sufficiently defined which again complicates comparison of data between different studies and laboratories. For instance, in some studies, lard (usually 15%) is used to induce an obese phenotype whereas in other studies coconut oil (about 20–30%) is administered [5]. In this regard, it is noteworthy that these two major fat sources do not only differ substantially in their composition, relevant variations are also observed between different lard and coconut oil batches [6]. Lard consists of approximately 40% saturated, 45% monounsaturated, and 15% polyunsaturated fatty acids whereby the three dominant fatty acids are palmitic acid, oleic acid, and stearic and linoleic acid. In contrast, coconut oil contains mostly saturated fatty acids (about 90%) and only minor amounts of monounsaturated and polyunsaturated fatty acids (about 6% and 2%, respectively). It is characterized by high amounts of lauric, myristic, capric, and caprylic acid which differ significantly from lard [6].

Accordingly, high-sugar diets comprise either variable amounts of glucose, fructose, or sucrose [5], which complicates inter-laboratory comparisons. Furthermore, protocols for energy restriction, known to affect the life and health span of model organisms, have not yet been standardized for experimental *D. melanogaster* research. For example, in the majority of fly studies focusing on dietary restriction, a protein/amino acid restriction has been provoked by a reduction in yeast [7], disregarding the fact that in most *Drosophila* diets yeast is also the sole source for other crucial nutrients. Differences in diet composition may also contribute to the high variance in the observed effects of energy restriction mimetics on life and health span in *D. melanogaster* [8, 9]. To overcome the limitations of complex diets, various attempts have been undertaken to create a semi-defined or fully defined medium for fruit flies [10–15]. Piper and coworkers [14] have established a holidic diet for *D. melanogaster*. This holidic diet is fully defined in terms of their energy, macro- and micronutrient composition. Most importantly, the chemically defined semisynthetic diet is supporting *Drosophila* development but as compared to complex diets it is characterized by a significantly reduced success rate and a drastically prolonged developmental time. Furthermore, the fecundity of flies raised on the holidic medium is considerably reduced when compared to complex media. Similar limitations have been reported for other semi-defined or fully defined diets [15]. Thus, the holidic diet may lack yet unidentified nutrients which are present in complex diets. Accordingly, only few studies address the exact fatty acid, vitamin, and trace element requirements of *D. melanogaster*. Therefore, future studies are needed which may improve the nutritional quality of holidic experimental diets.

### *Drosophila* phenotyping and diet-disease interactions

**Fig. 1:**
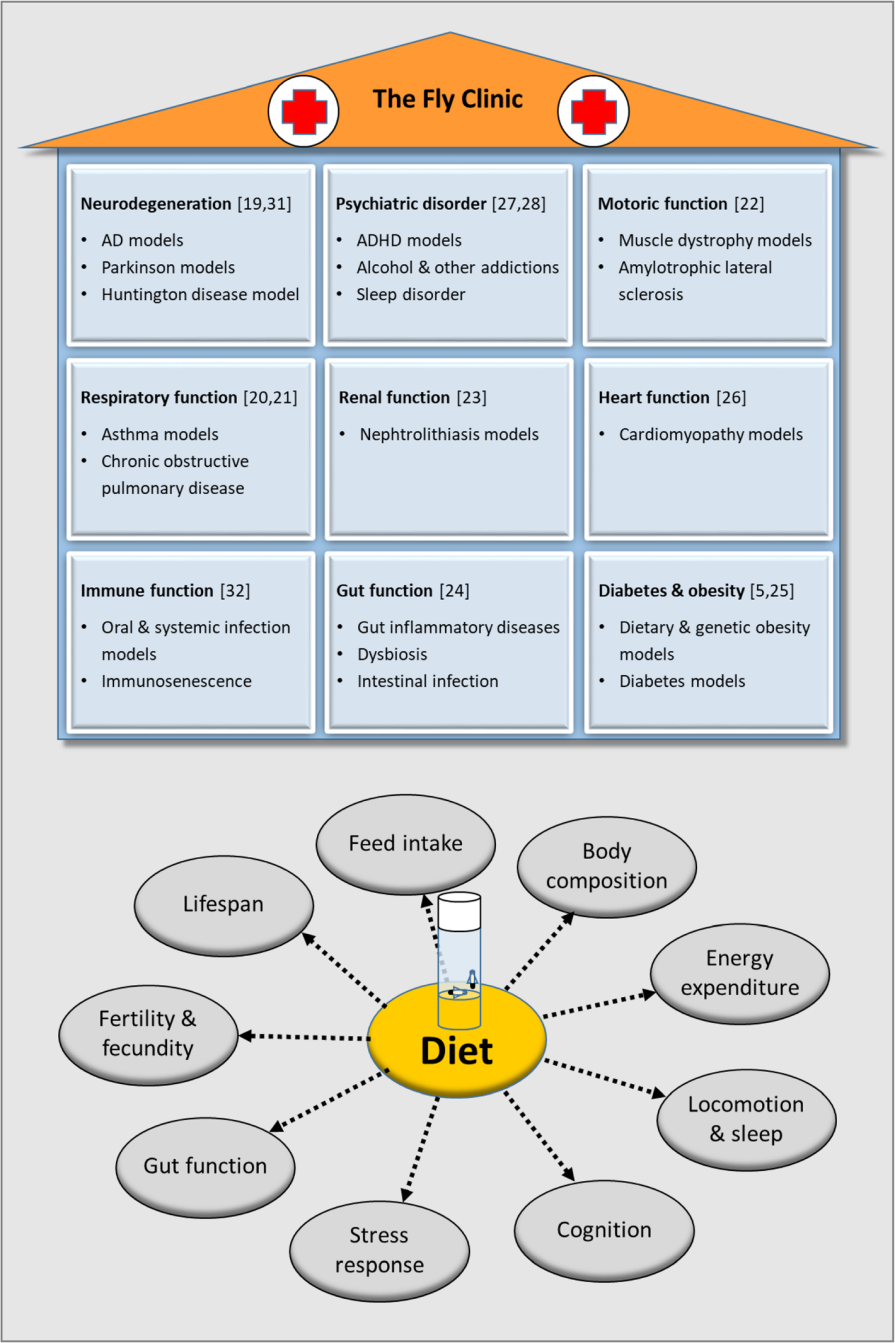
The fly clinic. Comprehensive phenotyping in *Drosophila melanogaster* forms the foundation of the fly clinic, where disease-related *Drosophila* models are employed to study diet-disease interactions [5, 19–28, 31, 32]

*D. melanogaster* can undergo a comprehensive phenotyping also in response to dietary factors. From a nutritional perspective, food intake, food choice, body composition, energy expenditure, and microbiota composition are important readouts [2]. These readouts are further complemented by other functional assays such as locomotor activity and sleep, cognition, stress and infection response, life span, and fertility depending on the experimental setting [2, 16, 17]. Thus, similar to laboratory mice, comprehensive phenotyping platforms are available for fruit flies as summarized in Fig. 1.

*D. melanogaster* enables also the possibility to conduct studies in disease-related models. Thus, there are various mutants as well as transgenic models available, which partly resemble chronic diseases prevalent in humans [17, 18]. In fact, *D. melanogaster* has been used to study pathologies related to brain function (A beta and tau pathology, Parkinson disease, Huntington disease) [19], respiratory function (asthma, chronic obstructive pulmonary disease (COPD)) [20, 21], motoric function (muscular dystrophy, amylotrophic lateral sclerosis) [22]), renal function (nephtrolithiasis) [23], gut disorders [24], diabetes [25], and heart function (cardiomyopathy) [26] as well as psychiatric disorders (ADHD, alcohol, and other addictions) [27, 28].

To study these complex and often multifactorial diseases in the fruit fly, two different approaches are applicable depending on the very nature of the disease: (i) Heterologous transgenic *D. melanogaster* models are employed to study key pathogenic proteins that are usually not present in the fly. A typical example for that are the neurodegeneration models, where, e.g., human Alzheimer’s disease genes (such as amyloid precursor protein, A-β peptides, or tau proteins), human Parkinson’s disease genes (α-synuclein, parkin), or polyQ disease genes are expressed in the fly. These animals have been successfully analyzed to assess biological effects and pathways involved in the disease process [18]. (ii) Homologous/analogous fly disease models are used to study evolutionary conserved disease genes that are found both in flies and humans. It has been estimated that about two thirds of human disease-causing genes have a functional homolog in the fly. A characteristic example for the second type of *Drosophila* models employing functional fly homologs is found in the field of lung disease research. Most susceptibility genes for complex lung diseases such as asthma have homologs in the fly [29], and it was possible to elucidate the functional role of the asthma susceptibility gene ORMDL3 using this approach [30]. We would like to emphasize here that although these fly models can be helpful to elucidate novel information about fundamental genetic and cellular processes underlying certain diseases, they are usually only able to model certain aspects of the abovementioned complex and multifactorial human diseases.

Disease mimicking *Drosophila* models may be subjected to different dietary regimens to single out diet-disease interactions. The ultimate goal of such studies is the identification of nutrients or dietary regimens that mitigate or accelerate the disease process. Diet-disease interactions have been already investigated in a limited number of fly studies. Parkinson’s disease models especially have been employed to identify novel nutrient- and diet-based therapy approaches. In particular, dietary factors like ascorbic acid, polyphenols, allyl disulfide, and sulforaphane as well as dietary zinc have been demonstrated to have positive effects in several different Parkinson’s disease fly models [31]. Other examples are studies on the impact of high-sugar or high-fat diets on heart health. The signaling and metabolic pathways that regulate the physiology of the fly heart show a remarkable high degree of conservation to the human heart. Hence, mutants and transgenes of the respective *Drosophila* genes have been used to investigate channelopathies and cardiomyopathies. Similar to the situation found in humans, where the metabolic syndrome is associated with an increased incidence of cardiomyopathies, high-sugar or high-fat diets led to increased arrhythmia and deterioration of the fly heart [26]. Thus, combining comprehensive phenotyping platforms with disease-related *Drosophila* models (in response to dietary factor) lays the foundation of establishing a so-called fly clinic (Fig. 1). Nevertheless, it has to be kept in mind that *Drosophila* disease-related models have merits and limitations. Thus, studies in *Drosophila* should be ultimately verified in other organisms of increasing biological complexity including mammalian species.

## Outlook and conclusion

Overall, it is suggested that a consensus within the scientific *Drosophila* community should be reached to standardize the exact composition of complex diets (including Western type and high-fat diets) for nutritional studies. Furthermore, also the composition of semisynthetic holidic diets (which may require further optimizations) should not vary between studies and laboratories. In order to exactly define the composition of holidic diets, additional studies addressing the fatty acid, vitamin, mineral, and trace element requirements of *D. melanogaster* may be needed in the future. Currently, it is unclear whether nutrient requirements vary between different fly strains (e.g., Oregon R versus Canton S versus w^1118^) and if nutrient demands are different between male and female as well as young and old flies. Finally, studies regarding the exact quantitative nutrient requirements for *Drosophila* maintenance, husbandry, and reproduction are also warranted.
